# First case of peritoneal cysticercosis in a non-human primate host (*Macaca tonkeana*) due to *Taenia martis*

**DOI:** 10.1186/1756-3305-7-422

**Published:** 2014-09-04

**Authors:** Julie Brunet, Bernard Pesson, René Chermette, Pierrick Regnard, Felix Grimm, Peter Deplazes, Xavier Ferreira, Marcela Sabou, Alexander W Pfaff, Ahmed Abou-Bacar, Ermanno Candolfi

**Affiliations:** Institut de Parasitologie et Pathologie Tropicale, EA 7292, Fédération de Médecine, Translationelle, Université de Strasbourg, 3 rue Koeberlé, 67000 Strasbourg, France; Faculté de pharmacie de Strasbourg, 74 route du Rhin, 67401 Illkirch, France; Service de Parasitologie-Mycologie & INRA, AFSSA, ENVA, UPVM, UMR 956 BIPAR, École Nationale Vétérinaire d’Alfort, 7, avenue du Général de Gaulle, 94704 Maisons-Alfort, Cedex France; Centre de Primatologie UdS - SILABE (Simian Laboratory Europe) ADUEIS, Fort Foch, 67207 Niederhausbergen, France; Institute of Parasitology, Vetsuisse Faculty, University of Zurich, Winterthurerstr. 266a, CH-8057 Zurich, Switzerland; Clinique vétérinaire des Halles, 28 rue du faubourg Saverne, 67000 Strasbourg, France

**Keywords:** *Taenia martis*, Cysticercosis, Zoonosis, *Macaca tonkeana*, France

## Abstract

**Background:**

Infections with larval stages (metacestodes) of a variety of taeniid species have been described in primates, including humans, with partial to severe clinical consequences. *Taenia martis* is a tapeworm of mustelids, and martens are mainly their definitive hosts in Central Europe. In the rodent intermediate host cysticerci develop in the pleural and peritoneal cavities. The present report describes a case of *T. martis* peritoneal cysticercosis in a Tonkean macaque.

**Findings:**

An abdominal mass was detected in a 3-year-old male Tonkean macaque (*Macaca tonkeana*) born and raised in a primate colony in France. Examination of the mass after laparotomy showed numerous vesicles identified as cysticerci of *T. martis*, based on the morphology of scolex and hooks, with confirmation by PCR amplification and sequence analysis of the mitochondrial cytochrome c oxidase subunit 1 (*cox1*) and NADH dehydrogenase subunit 1 (*nad1*) genes. Exeresis of the lesion was not possible and praziquantel (5.7 mg/kg) was given twice at an interval of 3 days. The abdominal mass was greatly diminished upon examination 2 months later and no signs of recurrence were noticed during the following 4 years.

**Conclusions:**

This is the first report of *T. martis* cysticercosis in a monkey. This record and the recent first description of an ocular *T. martis* cysticercosis in a human show the susceptibility of primates to *T. martis* and its zoonotic potential. This taeniid species must be considered in the differential diagnosis of cysticercosis in primates.

## Findings

Non-human primates might act as aberrant hosts for a number of cestode species after peroral infection and larval development in extra-intestinal locations can have potentially severe clinical consequences [1–6]. In most cases the circumstances of infection of captive monkeys remains unclear [3]. Tonkean macaques (*Macaca tonkeana*, Cercopithecidae) are housed in social groups in large enclosures, and are often used for ethological research [7]. *Taenia martis* (Zeder, 1803) is a tapeworm that develops in the small intestine of wild carnivores (Mustelidae). In Europe it is commonly found in *Martes foina,* the stone marten, and in *M. martes*, the pine marten. Other mustelids, and more rarely canids, might also act as definitive hosts [8]. The stone marten is established in the Northern Hemisphere and is widely found in France with the highest population densities reported in eastern regions [9]. *T. martis* has been observed in martens in Italy, Germany and Switzerland [10, 11] but there is a lack of information concerning its distributional range in France. However, cestodes found in *Martes foina* near Nancy and Paris, which were originally described as *T. intermedia* (Rudolphi, 1809), were later classified as *T. m. martis* [12, 13]. *T. martis* is maintained in a wild-animal cycle between carnivores and small rodents which act as intermediate hosts, mainly *Myodes* voles and *Apodemus* field mice. In Europe, prevalences of *T. martis* larvae in different rodents have been reported to vary between 0.95% in bank voles (*Clethrionomys* [currently *Myodes*] *glareolus*) in the French Pyrenees, 2% in *Apodemus flavicollis* in Switzerland, and 22% in musk rats (*Ondatra zibethicus*) in Belgium [11, 14, 15]. Recently, a first case of cysticercosis due to *T. martis* was observed in a human patient in Germany that shows the zoonotic potential of this cestode species [16]. The susceptibility of primates to *T. martis* infection is also supported by the present case of a peritoneal cysticercosis due to *T. martis* in a Tonkean macaque that, to the best of our knowledge, had never been reported before in a non-human primate.

**Figure 1:**
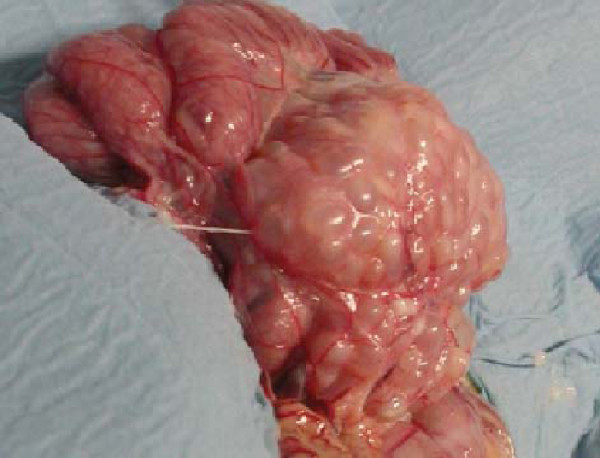
**Aspect of the lesion after celiotomy.** A vesicular mass embedded in the mesentery is shown. Some isolated cysticerci (5 to 30 mm long) are located at the right bottom.

**Figure 2:**
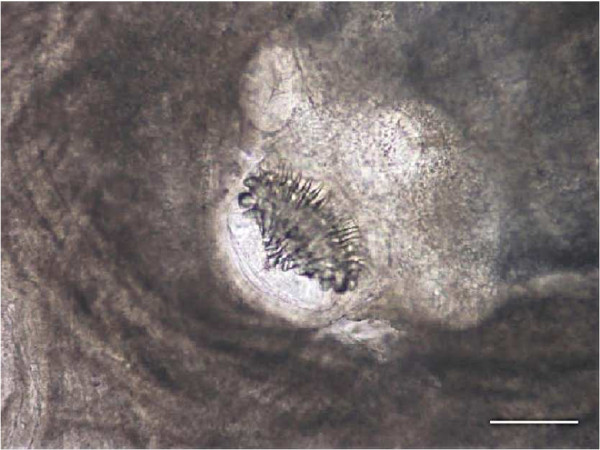
**Close-up appearance of the invaginated scolex inside a cysticercus.** Scale bar 250 μm.

**Figure 3:**
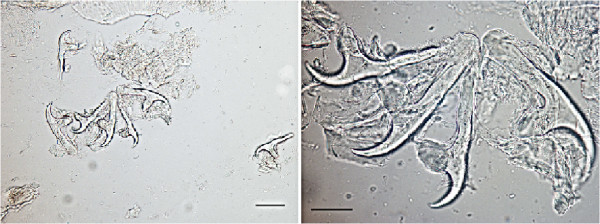
**Morphology of small and large rostellar hooks of** ***Taenia martis*** **from the present case (left: scale bar 100 μm; right: scale bar 50 μm).**

The animal was a subadult 3-year-old male Tonkean macaque with a weight of 5.2 kg, born and raised at the Strasbourg University Centre of Primatology, located near the Niederhausbergen forest (Alsace, northeastern France). The monkey was kept in a social group of 16 Tonkean macaques dedicated to ethological studies and housed in a semi free-range parkland closed by wire fencing. The animals feed on fruits and seeds provided by the Centre and on natural vegetation foraged in the park. Inside indoor-cages they have access to water and banana-flavoured monkey chunks *ad libitum*. A preventive anthelmintic treatment is administered twice a year (ivermectin 1 mg/3 kg subcutaneously, then fenbendazole 48 mg/kg orally, 6 months later). No abnormalities were reported for the infected animal until March 2009 when an abdominal mass (±10 cm × 5 cm) was detected at palpation during a routine check-up. No other clinical signs such as fever, diarrhea, anorexia or weight loss were observed. Blood tests revealed an increased alkaline phosphatase level (1610 UI/l) and a mild anaemia: haemoglobin 9.1 g/dl (reference range: 12.2 ± 0.8 g/dl), haematocrit 32.2% (40.3 ± 3.7%), erythrocytes 5.53 M/mm^3^ (6.5 ± 0.71 M/mm^3^). Other values were within normal range. Abdominal echography was performed and the lesion was visualized in the upper left part of the abdomen, obviously between the liver and the left kidney. After celiotomy, the mass appeared embedded in the mesentery (Figure 1). It contained hundreds of ellipsoidal to spherical transparent vesicles ranging from 5 to 30 mm in length. The vesicles had a white, opaque area at one pole and a tapering end at the opposite, some with an elongated extremity. No budding of daughter cysts was observed. They were identified as cysticerci at microscopic examination. Each cysticercus contained a single invaginated scolex (1 mm in diameter), with 4 suckers and 28 to 30 rostellar hooks arranged in two rows (Figure 2). The hooks had the same morphological aspect (upper edge nearly straight; handle thin, longer and in the same axis than the blade; blade short, rather abruptly curved; guard very prominent) and measured 140 to 160 μm and 180 to 212 μm in length for small and large hooks, respectively (Figure 3).

**Figure 4:**
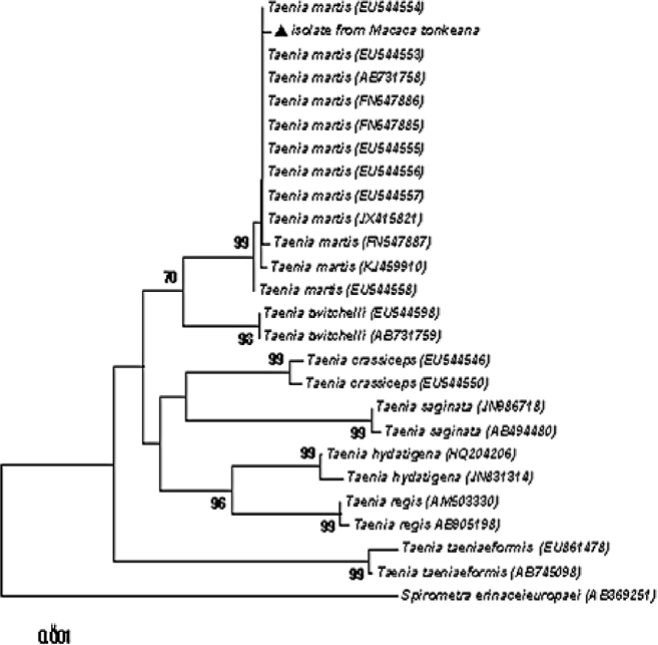
**Phylogenetic tree based on** ***Cox1*** **sequences showing the phylogenetic relationships between the** ***Taenia martis*** **isolate from** ***Macaca tonkeana*** **and other** ***Taenia*** **species.** Shaded triangle refers to the sequence generated by this study and the Genbank accession numbers of each strain are given into parenthesis. Bootstrap values >70% are included to indicate branch support.

Morphology of the cysticerci and rostellar hooks, and comparison with the closely related species *T. crassiceps*, led to the identification of *T. martis* [8, 17, 18]. Molecular tools confirmed the morphological identification. Sequences of the mitochondrial cytochrome c oxidase subunit 1 (*cox1*) and NADH dehydrogenase subunit 1 (*nad1*) genes were amplified by PCR with primer pairs JB3/JB4.5 and JB11/JB12 [19, 20]. The sequences showed identities of 99.8% (438/439 bp, *nad1*, GenBank accession number EU544606) and 99.7% (382/383 bp, *cox1*, AB731758) with sequences published for *T. martis*. Sequence homologies with the closely related species *T. twitchelli* and *T. crassiceps* were 94.0% (346/368 bp, EU544598) and 90.6% (347/383 bp, AF216699) for *cox1* and 86.8% (381/439 bp, EU544650) and 82.7% (363/439 bp, EU544600) for *nad1*, respectively. Phylogenetic tree was drawn by using the sequences obtained in this study as well as sequences available for representative *Taenia* species in GenBank (Figure 4). Diagnosis of peritoneal cysticercosis due to *T. martis* was therefore established. Surgical exeresis of the parasitic mass was not possible because of its localization and structure. Praziquantel (5.7 mg/kg, intramuscularly) was given twice at an interval of 3 days. Two months later, the abdominal mass was no longer palpable and the ultrasonography confirmed its dramatic decrease. No signs of recurrence were noticed during the 4-year follow-up period. The monkey exhibited normal growth and is presently healthy, and socializes perfectly within the group.

**Figure 5:**
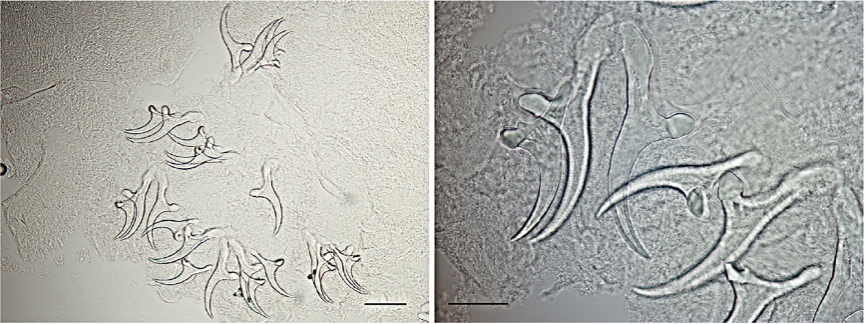
**Morphology of small and large rostellar hooks of** ***T. crassiceps*** **from a fox (left: scale bar 100 μm; right: scale bar 50 μm).**

The case of peritoneal cysticercosis caused by *T. martis* described herein in a Tonkean macaque, constitutes a first record of a metacestodosis in this mammalian host. Diagnosis of *T. martis* cysticercosis is difficult because clinical signs are not specific. Different etiologies were suspected for the abdominal mass detected in the macaque. Abdominal tumor and other metacestodoses, including alveolar echinococcosis (AE) were initially considered. AE has been described in captive monkeys including other *Macaca* spp., and *Echinococcus multilocularis* is endemic in the Alsace region where the animal lives [3, 6, 21]. The parasites sampled from the lesion were diagnostic for cysticercosis but identification of *Taenia* species is challenging due to the similarities of their morphological characteristics [22]. Cysticerci of *T. crassiceps*, *T. hydatigena* and *T. solium* have been described in non-human primates. In the current case, some larvae showed a strobila-like structure, which is commonly seen in *T. polyacantha* [23]. However, the size of the small hooks and, most importantly, the total number of hooks in the present case clearly distinguished it from *T. polyacantha* (112–148 μm and 62 hooks). Due to the size of the cysticerci and their high number, *T. crassiceps* infection was also suspected, but comparison of scolices and hooks with those of *T. crassiceps* collected from a fox and a dog, and the absence of budding cysticerci in our case, ruled out this hypothesis (Figure 5) [8, 17, 18].

In the present case, the completion of the cestode life-cycle is highly probable in the environment of the park as small rodents and carnivores including martens were regularly observed in the close vicinity. The infection of the monkey was likely through ingestion of taeniid eggs during foraging activities, which are quite developed in Tonkean macaques [7]. Taeniid eggs might contaminate soil and grass when scattered by rain from faeces of wild carnivores, which are often found near the fences or possibly inside the enclosure as access of martens to this wooded park is not excluded. Direct infection by picking the potentially contaminated faeces, especially when they contain fruit stones or during grooming from the fur of monkeys from the ground, are other hypotheses. In our case, clinical evolution was quite favourable. Although praziquantel had demonstrated a variable efficacy in the treatment of metacestodoses [17, 24], this cestodicidal drug was chosen because its injectable administration to the macaque was easier and more reliable than fenbendazole or albendazole, which would have required daily oral administration for several weeks. The localized infection observed in this macaque and the complete cure without surgical resection could be explained by a high sensitivity of *T. martis* larvae to praziquantel, by adequate host defenses (as no sign of immunosuppression was observed in the animal), and also by the absence of proliferation of the parasites. Indeed, the fully-developed cysticerci of *T. martis* are non-multiplying larvae contrary to those of *T. crassiceps* which proliferate by budding [8, 17, 25]. In the case of *T. martis* metacestodosis reported recently in an immunocompetent woman in southeastern Germany, the lesion was due to a single cyst localized in the eye and the cure was obtained by surgical removal of the parasite [16]. The present report shows the susceptibility of macaque to *T. martis* larvae and indicates the occurrence of an environmental contamination with *T. martis* eggs. It highlights the potential risk of infection for captive monkeys and also for humans with this taeniid species, which should be considered in the etiology of atypical cysticercosis cases.
